# Dysbiosis signatures of the microbial profile in tissue from bladder cancer

**DOI:** 10.1002/cam4.2419

**Published:** 2019-09-30

**Authors:** Fei Liu, Anwei Liu, Xin Lu, Zhensheng Zhang, Yongping Xue, Jinshan Xu, Shuxiong Zeng, Qiao Xiong, Haoyuan Tan, Xing He, Weidong Xu, Yinghao Sun, Chuanliang Xu

**Affiliations:** ^1^ Department of Urology Changhai Hospital Affiliated by the Senond Military Medical University Shanghai China; ^2^ Department of Urology Affiliated Hospital of Sergeant School of Army Medical University Shijiazhuang China

**Keywords:** 16s rRNA, bladder cancer, microbiota, urinary tract

## Abstract

**Background:**

To examine the microbial profiles in parenchyma tissues in bladder cancer.

**Methods:**

Tissue samples of cancerous bladder mucosa were collected from patients diagnosed with bladder cancer (22 carcinoma tissues and 12 adjacent normal tissues). The V3‐V4 region of the bacterial 16S rRNA gene was PCR amplified, followed by sequencing on an Illumina MiSeq platform. Bioinformatics analysis for microbial classification and functional assessment was performed to assess bladder microbiome diversity and variations.

**Results:**

The predominant phylum in both tissues was *Proteobacteria*. The cancerous tissues exhibited lower species richness and diversity. Beta diversity significantly differed between the cancerous and normal tissues. Lower relative abundances of the microbial genera *Lactobacillus*, *Prevotella_9,* as well as *Ruminococcaceae* were observed, whereas those of *Cupriavidus* spp., an unknown genus of family *Brucellaceae*, and *Acinetobacter*, *Anoxybacillus*, *Escherichia‐Shigella*, *Geobacillus*, *Pelomonas*, *Ralstonia*, and *Sphingomonas* were higher in the cancerous tissues. These findings indicate that these genera may be potentially utilized as biomarkers for bladder cancer. PICRUSt analysis revealed that several pathways involved in the metabolism of harmful chemical compounds were enriched in the cancer tissues, thereby providing evidence that environmental factors are strongly associated with bladder cancer etiology.

**Conclusion:**

This is the first study that has described and analyzed the dysbiotic motifs of urinary microbiota in the parenchymatous tissues of bladder cancer via *16S rRNA* gene sequencing. Our results suggest that changes in the bladder microbiome may serve as biomarkers for bladder cancer, possibly assisting in disease screening and monitoring.

## Graphical abstract

This is the first study describing and analyzing the dysbiosis signatures of urinary microbiota within the parenchyma in bladder cancer using a 16S rRNA gene sequencing method. The study reveals previously undescribed bacterial diversity present in human bladder. The bladder microbiome changes could be a biomarker of bladder cancer that could be used to help in screening for the disease. Analysis of the inferred bladder cancer metagenome identified the strongest association with the harmful chemical products that may be metabolized.

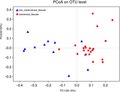

## 1 BACKGROUND

Urinary bladder cancer is the ninth most frequent malignant disease and the 13th most common cause of cancer‐related death around the world.^1^ It is the second most common urological malignancy and is responsible for 5% of all cancer‐related deaths. It accounts for US $4 billion worth of healthcare costs per year in the US alone. Indeed, 549 393 diagnosed cases of bladder cancer and 199 922 deaths due to bladder cancer were predicted to happen globally in 2018.^2^ The incidence increases with age and is relatively higher in males compared with females (3.2:0.9 ratio).^1^ Cigarette smoking is the most frequent risk factor for bladder carcinoma. In addition to cigarette smoking, exposure to aromatics, arsenic, hydrocarbons, and other environmental factors have been correlated with bladder cancer.^3^

New improvements in our comprehension of the composition have shown the crucial part that microbiota plays in the maintenance of health and development of disease. Unlike the rich diversity of bacterial species in other mucosal surfaces in the human body, the urinary tract is largely considered sterile, which may be attributed to the employment of culture‐dependent techniques for bacterial detection. Recent evidence has shown that dysbiosis of the urinary microbiota is closely related to many diseases of the urinary system, such as interstitial cystitis, urgency urinary incontinence, and neurogenic bladder dysfunction.^4^, ^5^, ^6^ More and more attention is being paid to the relationship between cancer and microbes, such as the relationship between *Helicobacter pylori* and gastric cancer and between human papillomavirus and cervical cancer.^7^, ^8^ Nevertheless, the correlation between these actively metabolizing microbial species and cancer remains unclear. At present, there is insufficient evidence linking the urinary microbiota and urothelial bladder carcinoma. A case‐control evaluation has revealed that the active intake of lactic acid bacteria decreases the risk for bladder cancer in the general human population.^9^ Another study has shown that the urinary microbiota of the urine may be strongly linked to bladder cancer.^10^ It is not yet clear whether the urinary microbiota of the cancerous tissue can become part of the tumor microenvironment associated with bladder cancer.

So far, no study has explored the relationship between the cancerous tissues and noncancerous tissue microbiome in bladder cancer. In order to study the microenvironment of bladder tumor tissues accurately, we examined the 16S rRNA genes to compare the microbial profile of bladder cancer tissues with normal tissues.

## 2 METHODS

### 2.1 Sample collection And DNA extraction

Mucosal tissue samples of bladder carcinoma were gathered intraoperatively from recently diagnosed patients [22 cancerous tissues and 12 nearby normal tissues (situated approximately 5 cm from the cancerous tissues)]. Twelve groups of cancer tissues and adjacent tissues were paired (samples are collected from the same individual but from two different sites: cancerous and adjacent normal tissues). The clinical parameters of the study subjects are presented in Table 1. The diagnoses were verified by biopsy as well as histological analysis. The subjects who fit any of the described exclusion criteria were removed from this evaluation, such as those with systemic diseases (such as immunodeficiency, cardiopathy, diabetes, or hypertension), received antibiotics in the past 2 months, and regularly treated with nonsteroidal anti‐inflammatory drugs, probiotics, or statins. Individuals who received preoperative chemotherapy or radiation therapy and those who had complications with acute/chronic infectious diseases of the bladder were also excluded from the study. The specimens were immediately brought to the laboratory (within 20 minutes) upon collection. DNA was isolated utilizing a Fast DNA SPIN extraction kit (catalog number: 116540600, MP Biomedicals, Santa Ana, CA, USA) and then kept at −80°C until analysis.

**Table 1:** Summary of information for the individuals in the study

	Cancerous tissues (n = 22)	Noncancerous tissues (n = 12)	*P*‐value
Gender, males/females	22/0	12/0	
Age, year	65 ± 8.7	66 ± 9.2	.7020
BMI, kg/m^2^	21.9 ± 1.4	22.2 ± 1.2	.5104
Pathological grade, low/high	5/17		
Biological characteristics, NMIBC/MIBC	5/17		

Abbreviations: BMI, body mass index; NMIBC, nonmuscle‐invasive bladder cancer; MIBC, muscle‐invasive bladder cancer.

### 2.2 16S rDNA microbial community analysis

PCR amplification of the V3‐V4 region of the bacterial 16S rRNA gene was conducted with the following primers: forward 338F (5'‐ACTCCTACGGGAGGCAGCA‐3') and reverse 806R (5'‐GGACTACHVGGGTWTCTAAT‐3'). The PCR of 25 μL components contained 5 μL of Q5 reaction buffer (5×, catalog number: M0491L, New England Biolabs, USA), 5 μL of Q5 High‐Fidelity GC buffer (5×, catalog number: M0491L, New England Biolabs, USA), 2 μL (2.5 mmol/L) of dNTPs (catalog number: Q5818, TIANGEN, China), 1 μL (10 μmol/L) of each Forward and Reverse primer, 3 μL of DNA Template, 0.25 μL of Q5 High‐Fidelity DNA Polymerase (5 U/μL, catalog number: M0491L, New England Biolabs, USA), and 7.75 μL of ddH_2_O. The PCR amplification was performed using the ABI 2720 PCR System with an initial denaturation at 98°C for 5 minutes; followed by 25 cycles consisting of denaturation at 98°C for 30 seconds, annealing at 52°C for 30 seconds, and extension at 72°C for 1 minute, with a final extension at 72°C of 5 minutes.

PCR amplicons were purified with Agencourt AMPure Beads (catalog number: AP‐GX‐500, Beckman Coulter, Indianapolis, IN), quantified using the PicoGreen dsDNA assay kit (catalog number: P7589, Invitrogen, Carlsbad, CA, USA), and sequenced using the Illumina MiSeq platform. Raw sequencing reads that exactly matched the barcodes were paired to respective samples and designated as valid sequences. These high‐quality sequences were grouped into operational taxonomic units (OTUs), showing 97% sequence identity. We selected a representative sequence from every OTU with the default parameters. An OTU taxonomic classification was performed using BLAST searching for the set of representative sequences against the Greengenes Database.^11^ Sequence data analyses were mainly performed using QIIME and R packages (v 3.2.0). Ace diversity index, Chao diversity index, Shannon diversity index, Simpson diversity index, and Sobs diversity index were calculated using the OTU table in QIIME. Beta diversity is used to illustrate changes in microbial community structure using Weighted UniFrac distance metrics and visualized via principal coordinate analysis (PCoA).^12^, ^13^ Variations in the Unifrac distances using pairwise comparisons among groups were assessed with the Student's *t* test as well as Monte Carlo permutation test using 1000 permutations. The significance of microbiota structural differentiation among groups was evaluated using permutational multivariate analysis of variance (PERMANOVA) with the adonis function as implemented in the R package “vegan”.^14^ Random forest (RF) analysis was utilized to distinguish samples from various groups with the R package “random Forest” using 1000 trees as well as default settings.^15^ The generalization error was calculated using a 10‐fold cross‐validation. We also included the expected “baseline” error that was established using a classifier that directly predicts the most frequent category label. To assess the discriminatory power of the RF model, we constructed operating characteristic curves (receiving operational curves, ROCs). We also predicted microbial functions using Phylogenetic Investigation of Communities by Reconstruction of Unobserved States (PICRUSt) with high‐quality sequences.^16^

### 2.3 Statistical analysis

The Student's *t* test was used to compare age and BMI between cancerous tissues and noncancerous tissues using the SPSS 21.0 software program for Windows (IBM) and the test was two‐tailed. Wilcoxon rank‐sum test was used to test the significant difference in the α‐diversity index between groups and the difference in the metabolic pathways of melanogenesis between cancerous tissues and noncancerous tissues using R packages (v 3.2.0). For correlation analysis, we conducted Spearman's rank test as implemented in the R packages (v 3.2.0). *P* < .05 was considered statistically significant.

### 2.4 Accession number

All raw sequencing data were deposited in the NCBI Sequence Read Archive under accession number SRP193356.

## 3 RESULTS

### 3.1 Sequence‐based characterization of the microbiome of cancerous tissues and noncancerous tissues

Analyses were performed on the samples with detectable bacterial DNA (for bladder cancer tissues, *n* = 22; for adjacent noncancerous bladder tissues, *n* = 12). A total of 1 954 443 valid sequences were obtained from 34 samples. After quality control of the sequencing data, a total of 34 samples obtained 1 534 433 high‐quality sequences, and the average sequence of each sample was 45,130. The number of specific sequences for each sample is shown in Table S1. To minimize variations in sequencing depth among samples, a table of the mean, rounded rarefied OTU values was constructed by computing for the average of 100 evenly resampled OTU subsets with minimum sequencing depths of <90% for further investigation. The sequences from these samples were classified into 36 phyla, 81 classes, 172 orders, 311 families, and 695 genera. The most abundant phyla detected were *Proteobacteria*, with abundance of 54.1%, followed by *Firmicutes* (23.7%), *Bacteroidetes* (13.4%), and *Actinobacteria* (4.4%). The most abundant genera detected were *Cupriavidus*, with an abundance of 16.9%, followed by unclassified *Brucellaceae* (6.0%), *Ralstonia* (5.5%), and *Lactobacillus* (5.3%).

### 3.2 Alpha and beta diversity between cancerous and noncancerous tissues

**Figure 1:**
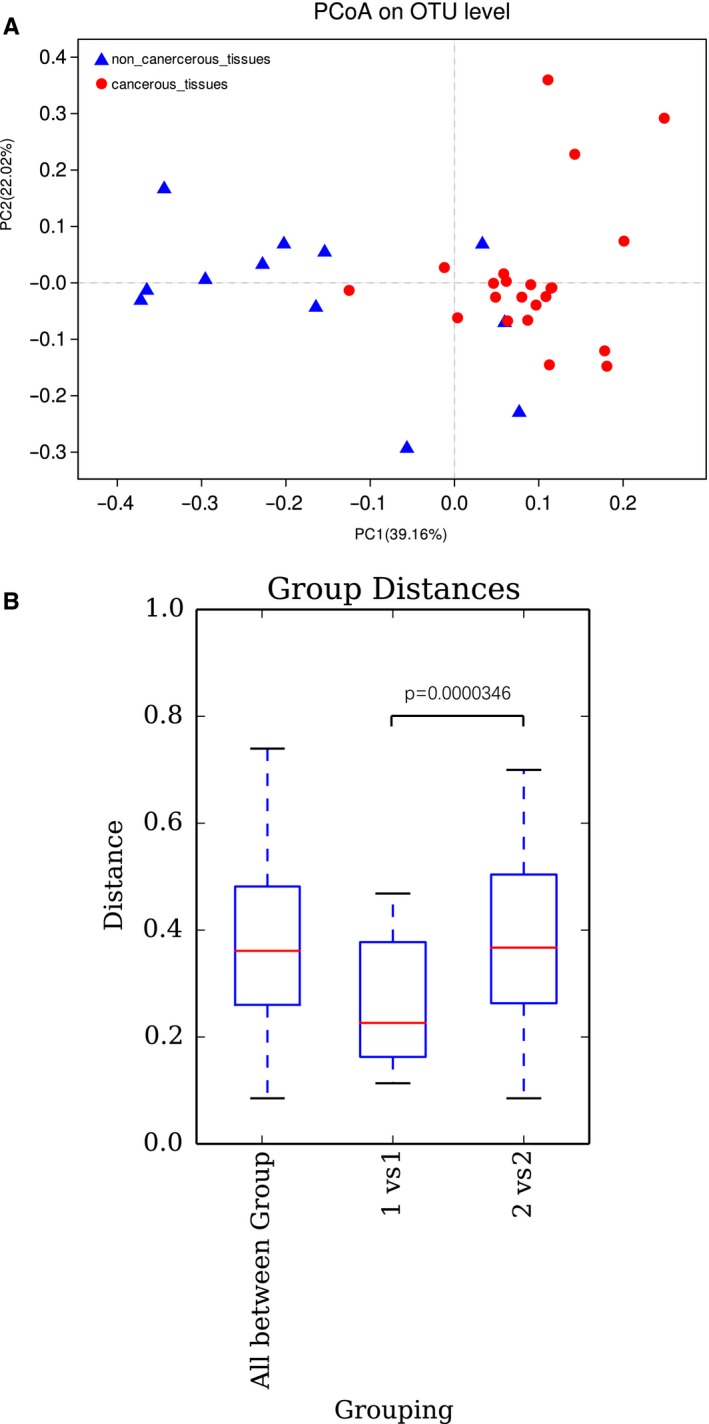
(A) Principal coordinate analysis (PCoA) of a Weighted UniFrac distance analysis. (B) Weighted Unifrac distance analysis of 12 pairs of cancerous and noncancerous tissues. The abscissa represents the comparison of samples between different groups (cancer tissues and noncancerous tissues) and within each group, and the longitudinal coordinates represent distance values of differences. The reliability of statistical analysis is expressed by *P*, and *P* < .05 is significant. All between groups refer to the difference between different individuals (cancer tissues and noncancerous tissues), 1 vs 1 refers to the difference between cancer tissues, and 2 vs 2 refers to the difference between noncancerous tissues. Box plot elements: the line inside, median; box limits, upper and lower quartiles; whiskers, 1.5× interquartile range

A total of 2606 OTUs in all samples were identified. We examined the α‐diversity indices of Ace, Chao, Shannon, Simpson, and Sobs in all 34 bladder tissue samples. A statistically significant difference in the Shannon diversity index was observed between cancerous and noncancerous tissues (*P* = .0417), demonstrating a significantly lower diversity in cancerous tissues compared to noncancerous tissues. Although no statistically significant differences were observed among the Ace, Chao, Simpson, and Sobs indices, which may be attributable to the low statistical power or variability within each group, lower degrees of species richness and diversity were observed in cancer tissues compared to noncancerous tissues (Figure S1A–E). Significant differences were detected in β‐diversity using the Weighted UniFrac distance statistic (qualitative, ADONIS *P* < .001) between cancerous tissues and noncancerous tissues, indicating that the mucosa‐associated microbial structure in the cancerous tissue group was significantly different from that of the noncancerous tissue group in the presence of OTUs (Figure 1A). Difference analysis based on weighted Unifrac distance showed that the inter‐individual variability of cancer tissues was significantly smaller than that of adjacent tissues in the 12 pairs (*P* < .001), and the results are shown in Figure 1B.

### 3.3 Alteration in the taxa between cancerous tissue and noncancerous tissue groups

**Figure 2:**
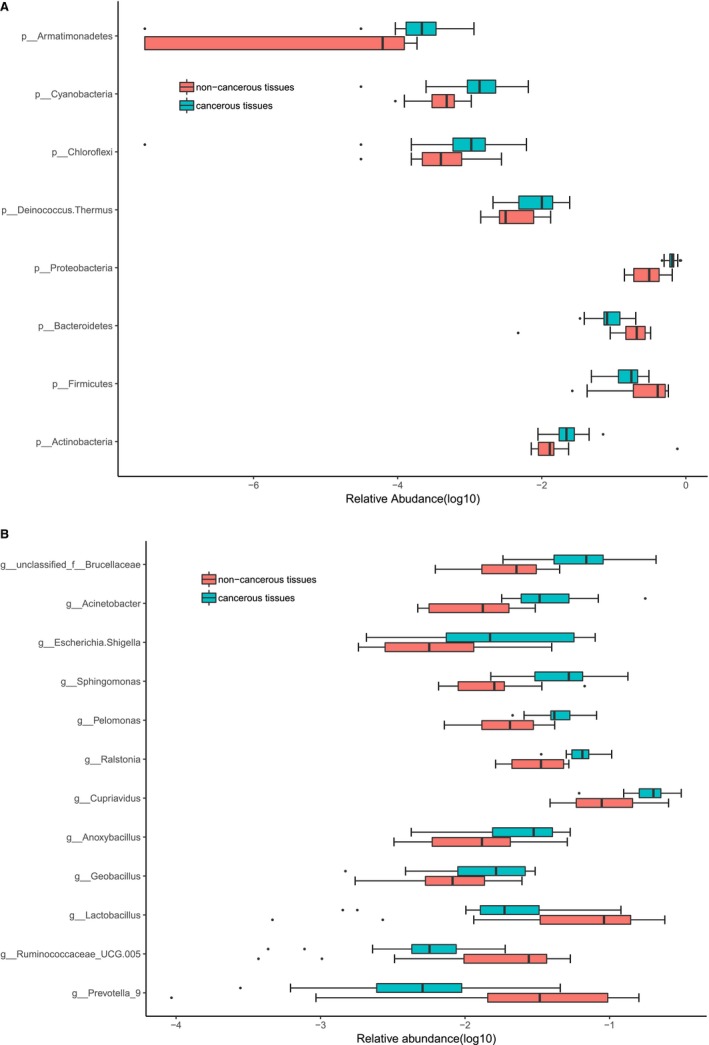
Microbiome modifications at the phylum and genus levels. (A) The relative abundance of eight phylum significantly varied among cancerous tissues and noncancerous tissues. (B) The relative abundance of 12 genera significantly varied between cancerous tissues and noncancerous tissues. Box plot elements: the line inside, median; box limits, upper and lower quartiles; whiskers, 1.5× interquartile range; points, outliers

To identify particular alterations in the microbiota in various bladder cancer samples, we examined the relative abundance of these taxa in cancer and normal tissues. At the phylum level, *Firmicutes* and *Bacteroidetes* were significantly decreased in cancerous tissues, whereas *Proteobacteria spp* and *Actinobacteria spp* were overrepresented in cancerous tissues (Figure 2A). At the genus level, approximately 12 bacterial taxa displaying different degrees of abundance between cancer and normal tissues were detected. *Lactobacillus*, *Prevotella_9, and Ruminococcaceae* were enriched in noncancerous tissues. Conversely, *Cupriavidus spp, unclassified Brucellaceae, Acinetobacter, Escherichia‐Shigella, Sphingomonas, Pelomonas, Ralstonia, Anoxybacillus*, and *Geobacillus* were significantly increased in cancerous tissues (Figure 2B).

**Figure 3:**
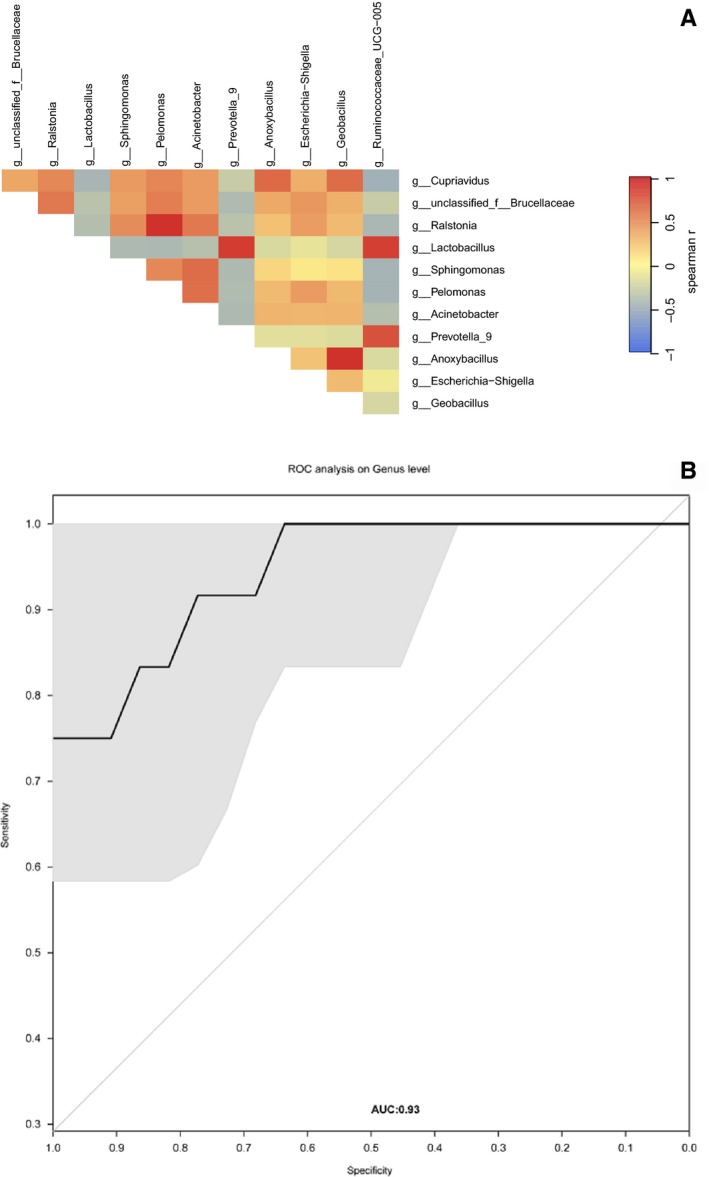
(A) Spearman correlations between the 12 genera in cancerous and noncancerous tissues. In general, microbial genera enriched in cancerous tissues (in red text) were negatively connected with noncancerous tissue‐enriched genera (in blue text). Strong correlations were observed between *Geobacillus* and *Anoxybacillus* (*R* = .9410); *Ralstonia* and *Pelomonas* (*R* = .9438). (B) The urinary microbiota signature can be utilized to differentiate between cancerous tissues and noncancerous tissues. Receiver operating characteristic curve analysis was performed on the exploratory data (area under curve (AUC) = 0.93)

Spearman correlation tests were conducted to assess the relationship among various bladder cancer‐associated genera. The analysis was based on 12 different microflora in bladder cancer and adjacent normal tissues. The correlation analysis was run using counts data. We observed significant positive correlations among the following bladder cancer‐associated genera: *Geobacillus* and *Anoxybacillus* (*R* = .9410); *Ralstonia* and *Pelomonas* (*R* = .9438). Generally, the genera that predominated cancerous tissues were negatively correlated with those that were abundant in the control subjects (Figure 3A), thereby indicating an antagonistic association among harmful and beneficial bacteria.

### 3.4 Random forest (RF) predictive models

To investigate the capacity of the bladder mucosa microbiome to distinguish bladder cancer status, we developed a RF model using the bladder mucosa microbiome motif, which is made up of 12 bladder‐associated genera. We then assessed the performance of the model via ROC analysis, which generated an area under curve (AUC) value of 0.93 (Figure 3B).

### 3.5 Association between clinical variables and the bladder mucosa microbiome

**Figure 4:**
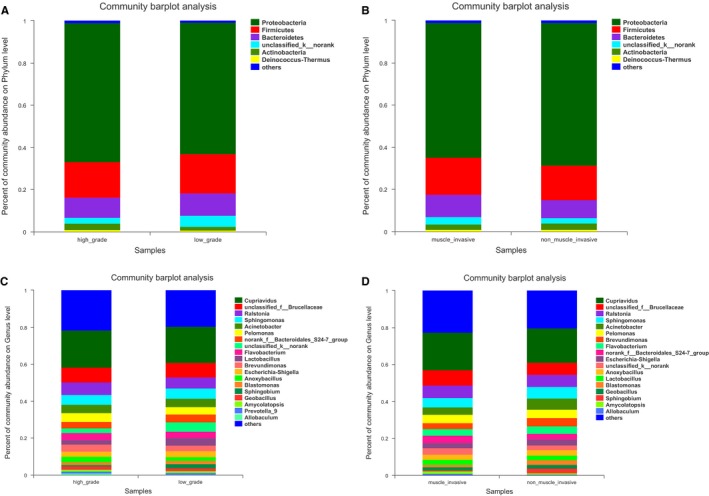
Different structures of urinary microbiota between low‐ and high‐grade, muscle‐invasive and nonmuscle‐invasive bladder cancer. (A) Relative urinary microbiota abundance at the phylum level of low‐ and high‐grade bladder cancer. (B) Relative urinary microbiota abundance at the phylum level of muscle‐invasive bladder cancer and nonmuscle‐invasive bladder cancer. (C) Relative urinary microbiota abundance at the genus level of low‐ and high‐grade bladder cancer. (D) Relative urinary microbiota abundance at the genus level of muscle‐invasive bladder cancer and nonmuscle‐invasive bladder cancer

We investigated the effects of sub‐phenotypes on the bladder mucosa microbiome in bladder cancer of different pathological grades and different biologically relevant subtypes. There were no significant differences in patients at different grades and different biologically relevant subtypes with respect to the α‐diversity or β‐diversity‐associated bladder cancer taxa. At the phylum level, *Proteobacteria* was the most predominant phylum in both of the groups, followed by *Firmicutes*. *Bacteroidetes, Unclassified_k_norank bacteria,* and *Actinobacteria* constituted the next most dominant phyla (Figure 4A,B). At the genus level, *Cupriavidus* was the most predominant genus in both of the groups, followed by *unclassified Brucellaceae*, *Ralstonia, Sphingomonas, Acinetobacter*, and *Pelomonas* (Figure 4C,D).

### 3.6 Predictive function analysis

We predicted the functional composition profiles from 16S rRNA sequencing data with PICRUSt (Phylogenetic Investigation of Communities by Reconstruction of Unobserved States) in the tissues. We observed a disruption in various KEGG (level 3) categories in bladder cancer. The enriched pathways of the bladder cancerous tissues were characterized by metabolism of harmful chemical products (eg, dioxin degradation, chloroalkane and chloroalkene degradation, naphthalene degradation, 1,1,1‐trichloro‐2,2‐bis (4‐chlorophenyl) ethane DDT degradation, nitrotoluene degradation, aminobenzoate degradation, and xylene degradation), some of which involve recognized carcinogens (eg, dioxin, nitrotoluene, aminobenzoate, and xylene). It is worth noting that multiple pathways related to pesticide metabolism were altered in the bladder cancerous metagenomes (eg, 1,1,1‐trichloro‐2,2‐bis (4‐chlorophenyl) ethane DDT degradation, nitrotoluene degradation, atrazine degradation, chlorocyclohexane and chlorobenzene degradation, and fluorobenzoate degradation).

**Figure 5:**
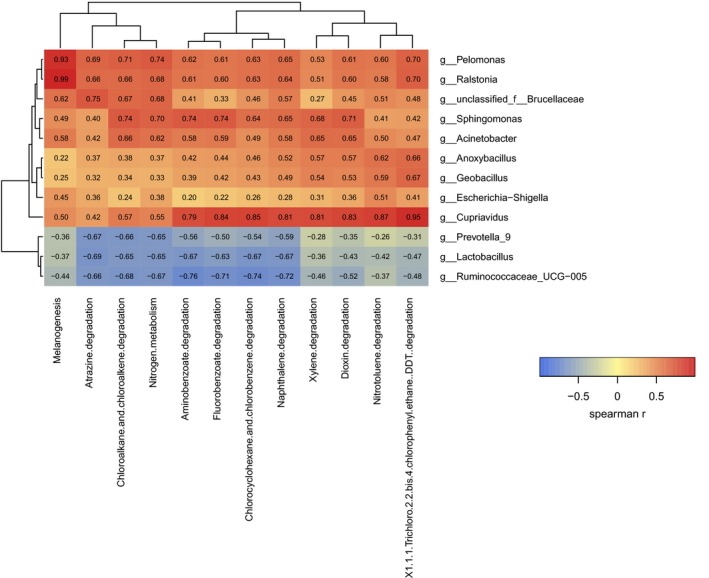
Heatmaps showing correlations between KEGG categories and urinary microbiota. The strength of the color depicts the r value (correlation) (negative score, blue; positive score, red)

**Figure 6:**
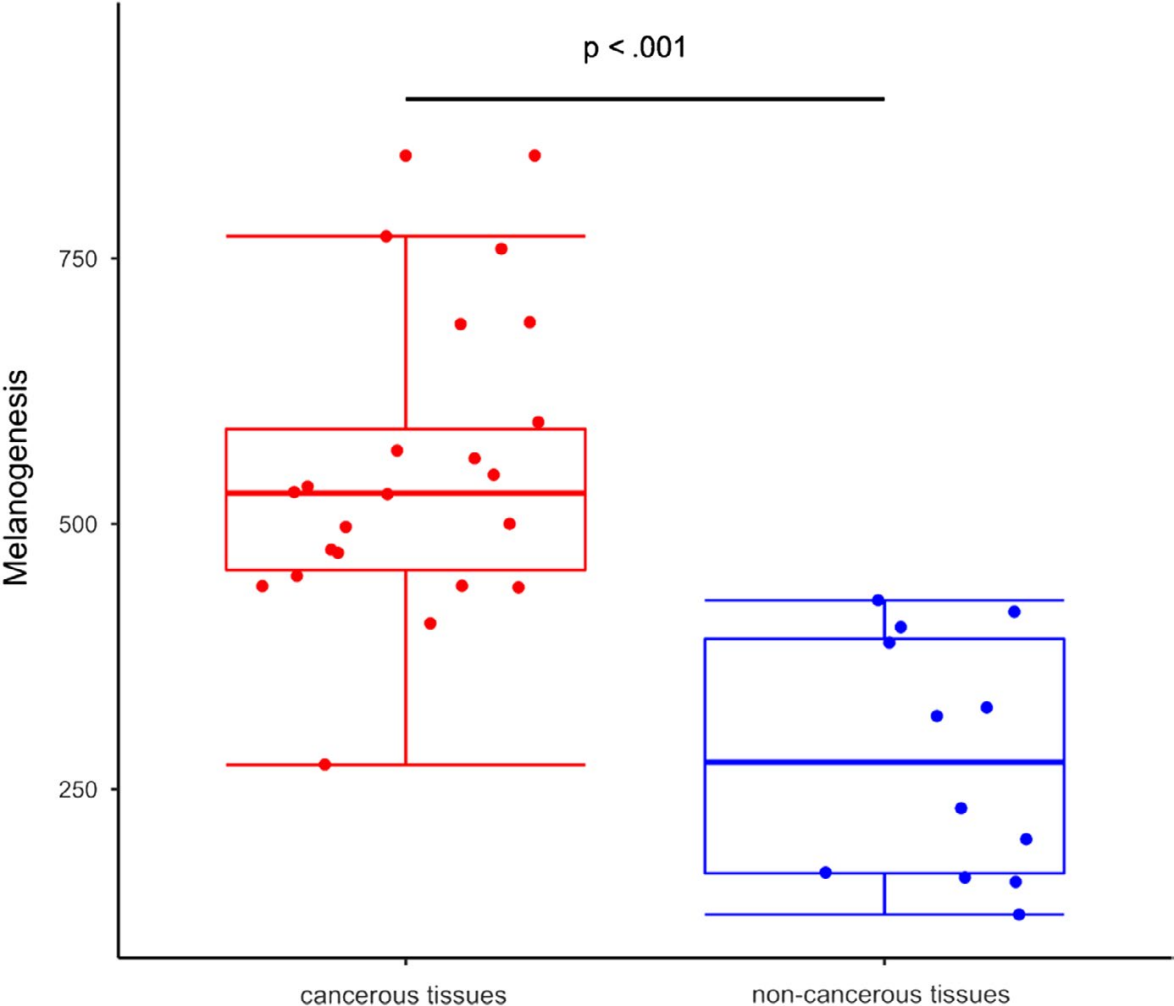
The predicted functional module, melanogenesis, altered in bladder cancer. A significant occurrence of differentially abundant melanogenesis pathway between cancerous tissues and noncancerous tissues. Box plot elements: the line inside, median; box limits, upper and lower quartiles; whiskers, 1.5× interquartile range; points, outliers

We selected 12 different metabolic pathways between cancer and normal tissues that were related to harmful substances and analyzed the correlation with the previous 12 different bacteria between cancer and normal tissues (Figure 5). The correlation analysis is based on counts data. Melanogenesis showed the most significant alterations among the predicted functional composition profiles (*R* = .9911; Figure 6).

## 4 DISCUSSION

The present evaluation conducted urinary microbial profiling of bladder cancer via 16S rRNA gene sequencing. Different from previous studies using urine specimens, we used bladder mucosa tissue as the study material. This is the microenvironment of bladder tumors and it provides the optimal specimens for the characterization of the bladder‐associated microbiome. We found that the Shannon diversity index, which measures both richness and evenness in the cancerous tissue group, was significantly lower than in the noncancer tissue group, suggesting a significantly lower degree of bacterial diversity in cancer tissues. Although other indexes did not show statistically significant differences, lower species richness and diversity were observed in cancerous tissues. Significant differences were observed in β‐diversity between cancerous tissues and noncancerous tissues in our study. In sum, both α‐ and β‐diversity data generate evidence that the urinary microbiota in bladder cancer tissues varies from those of normal bladder tissues.

Weighted Unifrac distance analysis indicated minimal differences in the structure of the microflora among tumor tissues, which were consistent with the observed lower level of species richness and diversity in tumor tissues using α‐diversity analysis. This result may be due to the predominance of pathogenic bacteria in cancer tissues.

Loss of microbiota diversity has recently been described as the most constant finding of intestinal dysbiosis in digestive diseases, such as irritable bowel syndrome (IBS), Crohn's disease, and colorectal cancer,^17^, ^18^ and in nondigestive diseases, such as Parkinson's disease.^19^ Changes in microbiome diversity have not been consistently associated with urinary tract diseases. Higher microbial diversity was detected in urgency urinary incontinence, and reduced diversity was found in overactive bladder.^20^, ^21^ In bladder cancer research, using urine specimens, one study observed increased richness of microbiota diversity in bladder cancer patients, whereas no significant differences in microbial diversity were observed in a different evaluation.^10^, ^22^ Based on our findings, we hypothesize that the loss of microbiota diversity may be related to the incidence of bladder cancer. As previously shown in the gut, the balance between specific microbial groups may be more relevant than overall urine composition. The balance of the urinary microbial ecosystem may be associated with the patient's lifestyle or other unknown factors. The gut microbial ecosystem and the total microbial biomass and abundance present in the urinary tract deserve further intensive study.

PCoA analysis revealed a significant differentiation in bacterial community composition among bladder cancerous tissues and noncancerous tissues. The improved understanding of the microbiome and cancer makes us recognize that the tumor‐promoting impacts of the colorectal cancer microbiota are apparently induced by modifications in the host‐microbiota interactions as well as by dysbiosis, instead of infections by specific pathogens.^23^ Changes in the urinary microbiome could also be related to bladder cancer development and progression. Novel insights may facilitate the prediction of bladder cancer risk as well as enable the creation of novel therapeutic strategies.

The relative abundances of the dominant phyla *Proteobacteria*, *Firmicutes*, and *Bacteroidetes* were all significantly different between cancerous tissues and noncancerous tissues. A significantly greater *Proteobacteria* abundance was found in cancerous tissues than in noncancerous tissues. The *Proteobacteria* are considered as gut commensals that possess features of pathogenicity. The dysbiosis that occurs during metabolic disorders, such as diet‐induced obesity, genetically induced obesity, and genetically induced diabetes, frequently includes higher prevalence for *Proteobacteria*.^24^, ^25^, ^26^ A recent study demonstrated obvious differences in the abundance of *Proteobacteria* in the mucosa‐related microbiome of ileal and rectal biopsies (but not in stool samples) among Crohn's disease patients and control subjects.^27^ Changes in the gut *Proteobacterial* community have also been reported in human colitis‐associated colorectal cancer.^28^ The results of the abovementioned analysis suggest that it is possible that an imbalance of *Proteobacteria* might play an important role in bladder carcinogenesis. An elevation in the prevalence of *Proteobacteria* may thus be a possible diagnostic signature for dysbiosis which may increase the risk of bladder cancer.

At the genus level, *Lactobacillus*, *Prevotella_9*, *Acinetobacter*, and *Escherichia‐Shigella* all displayed significant differences in abundance between cancerous tissues and noncancerous tissues. *Lactobacillus* and *Prevotella_9* were enriched in noncancerous tissues, while *Acinetobacter* and *Escherichia‐Shigella* were significantly increased in cancerous tissues.

*Lactobacillus* is an extensively examined probiotic bacterium that has the ability to promote a healthy body using a wide range of mechanisms. This includes colonizing resistance, generating acid, and competitively excluding pathogens.^29^ *Lactobacillus* has been detected in urine samples from patients with multiple urinary tract diseases such as urgency urinary incontinence and interstitial cystitis.^4^, ^5^ *Lactobacillus* was also found to be related to cancer. Cell‐free supernatants from *Lactobacillus* have been shown to decrease the invasion ability of a metastatic tumor cell line in vitro.^30^

In our study, a higher abundance of *Prevotella _9* was observed in both groups. *Prevotella* was also identified in urine samples of various urinary tract diseases. Emerging studies have linked the increased abundance of *Prevotella* species to chronic inflammation. Furthermore, the abundance of *Prevotella* was higher in the gut microbiome of colorectal cancer patients.

*Acinetobacter* was found in higher abundance in bladder cancerous tissues than other tissues. A recent study using urine samples reported that *Acinetobacter* occurred in higher abundance in bladder cancer patients compared to healthy controls.^10^ *Acinetobacter* is a genus that is considered to include pathogens that are important due to their multidrug resistance associated with significant mortality. It has been reported that *Acinetobacter* plays a significant role in disseminated infections, including in the respiratory and urinary tracts.^31^

It should also be noted that *Escherichia‐Shigella* was found in a higher abundance in cancerous bladder tissues than other tissues. *Escherichia‐Shigella* can cause inflammation of the colon mucosa, resulting in a series of symptoms such as diarrhea, abdominal pain, and mucosanguineous stool. *Shigella* infection of macrophages and epithelial cells induces a strong inflammatory response and macrophage death. It is necessary to test the function of *Escherichia‐Shigella* in bladder cancer.

The present study characterized bladder microbial dysbiosis based on changes in the abundance of 12 genera. Using these 12 differentiated taxa, cancerous tissues and normal tissues can be separated perfectly. The microbial signature that we identified may be possibly utilized in bladder cancer prediction. No statistically significant differences at different pathological grades or biologically relevant subtypes with respect to α‐diversity or β‐diversity associated bladder cancer taxa were observed with our current sample size. Whether any microbial dysbiosis exists between different pathological grades or biologically relevant subtypes in bladder tissues is worthy of further study. In this study, we did not analyze the correlation between the clinical indicators and the composition of bacterial flora in patients with bladder cancer. We analyzed only the differences in the composition of bacterial flora in different clinical types of bladder cancer. In the follow‐up study, we will further analyze the relationship between clinical indicators and the composition of bacterial flora in patients with bladder cancer.

Environmental factors are closely related to the incidence of bladder cancer. Drinking water or eating food that is contaminated with arsenic and ambient air pollution, which comes directly from combustion‐related chemicals, was documented to affect bladder cancer risk. In our study, a phylogenetic investigation of communities identified the strongest association with the harmful chemical products that may be metabolized. We particularly noted that multiple pathways related to pesticide metabolism were altered in the bladder cancerous metagenomes (eg, 1,1,1‐trichloro‐2,2‐bis (4‐chlorophenyl) ethane DDT degradation, nitrotoluene degradation, atrazine degradation, chlorocyclohexane and chlorobenzene degradation, and fluorobenzoate degradation).

Pesticides have been shown to lead to human cancer. For example, the pesticides that have alkyl ureas or amines are connected to brain tumors; dieldrin induces tumorigenesis of the lung, liver, lymphoid tissues, uterus, as well as thyroid; and the risk for prostate cancer is higher in patients exposed to Agent Orange.^32^, ^33^, ^34^ A prior investigation revealed that a trivalent pesticide‐related chemical can cause protein carbonylation and oxidative DNA damage in human urothelial cells, ultimately resulting in bladder cancer.^35^

Intriguingly, the enrichment of harmful chemical products, that may be metabolized, may be attributable to the significant elevation in the abundance of the genera *Cupriavidus*, which have the highest abundance of the bacteria in both bladder cancerous tissues and noncancerous tissues, although there are significant differences in abundance between them. *Cupriavidus* has been isolated as an organophosphorus pesticide‐degrading microorganism in previous studies. We infer that *Cupriavidus* decomposes the harmful substances absorbed by the body and then excretes these into the bladder through certain enzymes.

Analysis of the inferred bladder cancer metagenome identified the strongest association with the melanogenesis pathway. The possible role of melanogenesis in the pathogenic process of bladder cancer is unknown. Microorganisms commonly secrete melanin production to adapt to their immediate environment. Melanin production in pathogenic bacteria is strongly associated with virulence. The observed high melanin metabolic rates in bladder cancer tissues in this study may thus indicate enhanced melanin production by pathogenic bacteria in bladder cancer tissues, although the underlying pathogenic mechanism related to melanin production in bladder cancer tissues requires additional investigation.

PICRUSt is only a predictive tool. In order to obtain the accurate functional information of the related bacteria in samples, it is necessary to carry out metagenomics research in the future.

## 5 CONCLUSIONS

The present investigation verified the occurrence of bladder microbiota dysbiosis in bladder cancer. This study has revealed previously undescribed bacterial diversity present in the human bladder. As specific bacteria are associated with this body site, we infer that such bacteria may carry the potential to influence the development of pathophysiological processes in bladder cancer. We employed prediction models to detect differentially expressed bacterial taxa from bladder cancerous tissues and noncancerous tissues with high accuracy. The findings of this evaluation indicate that bladder microbiota may potentially be utilized as therapeutic targets and diagnostic biomarkers for bladder cancer. Analysis of the inferred bladder cancer metagenome identified the strongest association with the harmful chemical products that may be metabolized, which further confirmed that environmental factors are closely related to the incidence of bladder cancer. Determining the basis for these variations in bladder microbiota may offer new insights and better understanding of the pathogenesis of bladder cancer, as well as novel methods for its diagnosis and treatment. The small number of samples in the two groups is a limitation of this study. In the follow‐up study, we plan to further expand the sample size in order to obtain more accurate data.

## CONFLICTS OF INTEREST

All of the authors declare that there is no conflict of interest.

## ETHICS APPROVAL AND CONSENT TO PARTICIPATE

This study is in line with the Helsinki Declaration and was conducted in agreement with the guidelines of the Ethics Committee of the Changhai Hospital affiliated with the Second Military Medical University. All patients have written informed consent.
