# Supplementary figures and images for: Dysbiosis signatures of the microbial profile in tissue from bladder cancer
Source: Cancer Med. 2019 Sep 30;8(16):6904–14. doi: 10.1002/cam4.2419 (PMC6854010; doi:10.1002/cam4.2419)

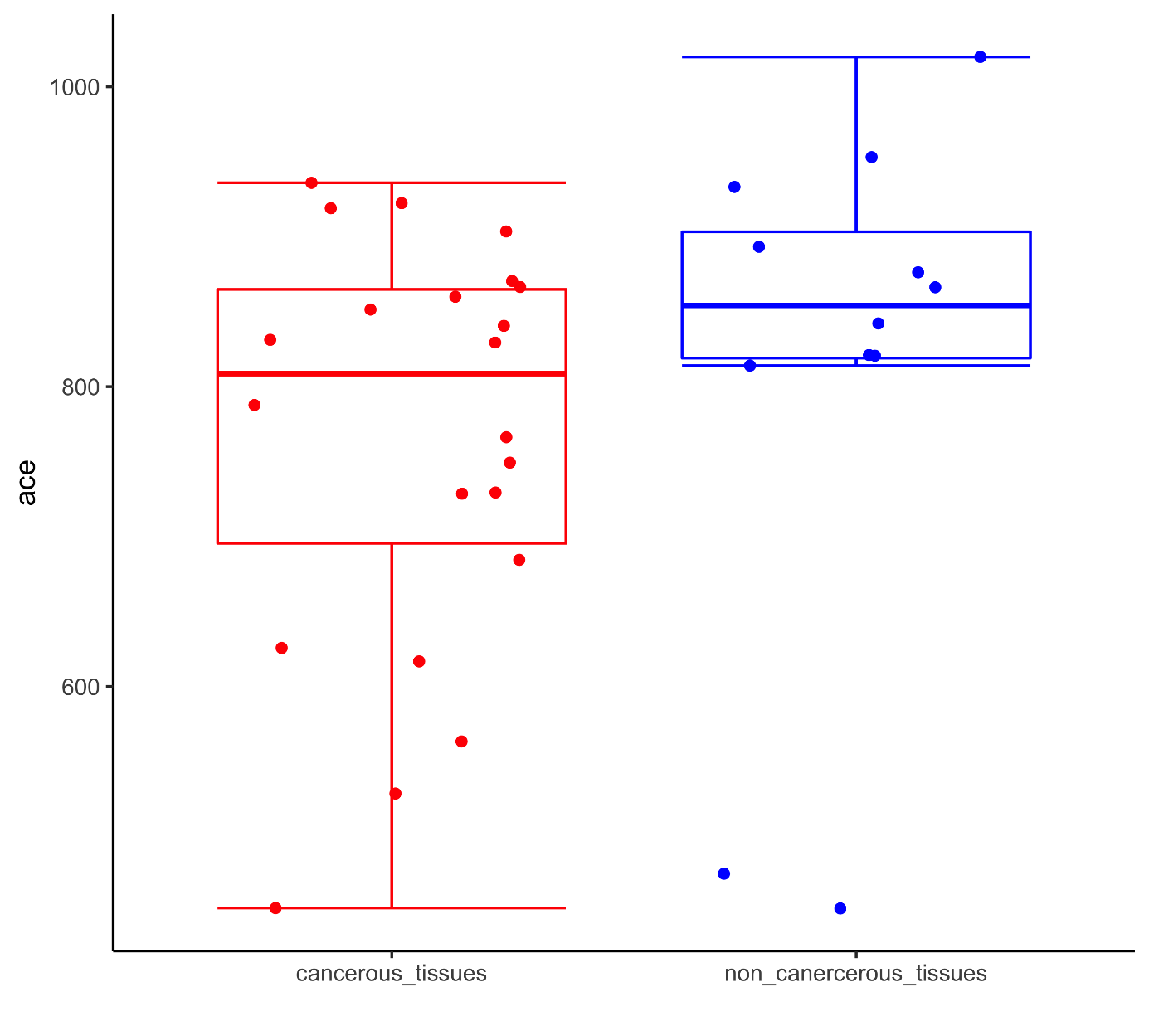


Figure.S1A


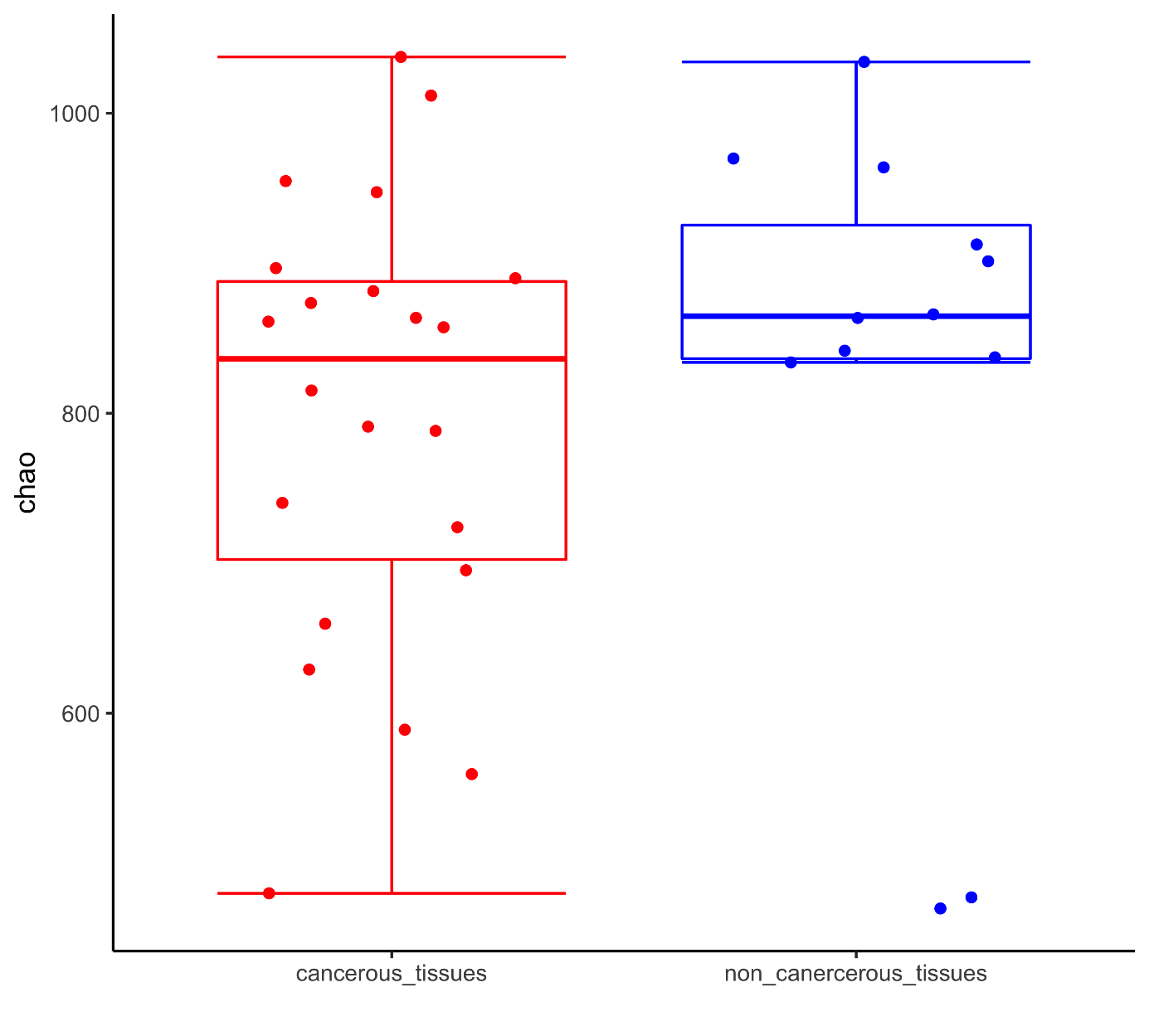


Figure. S1B


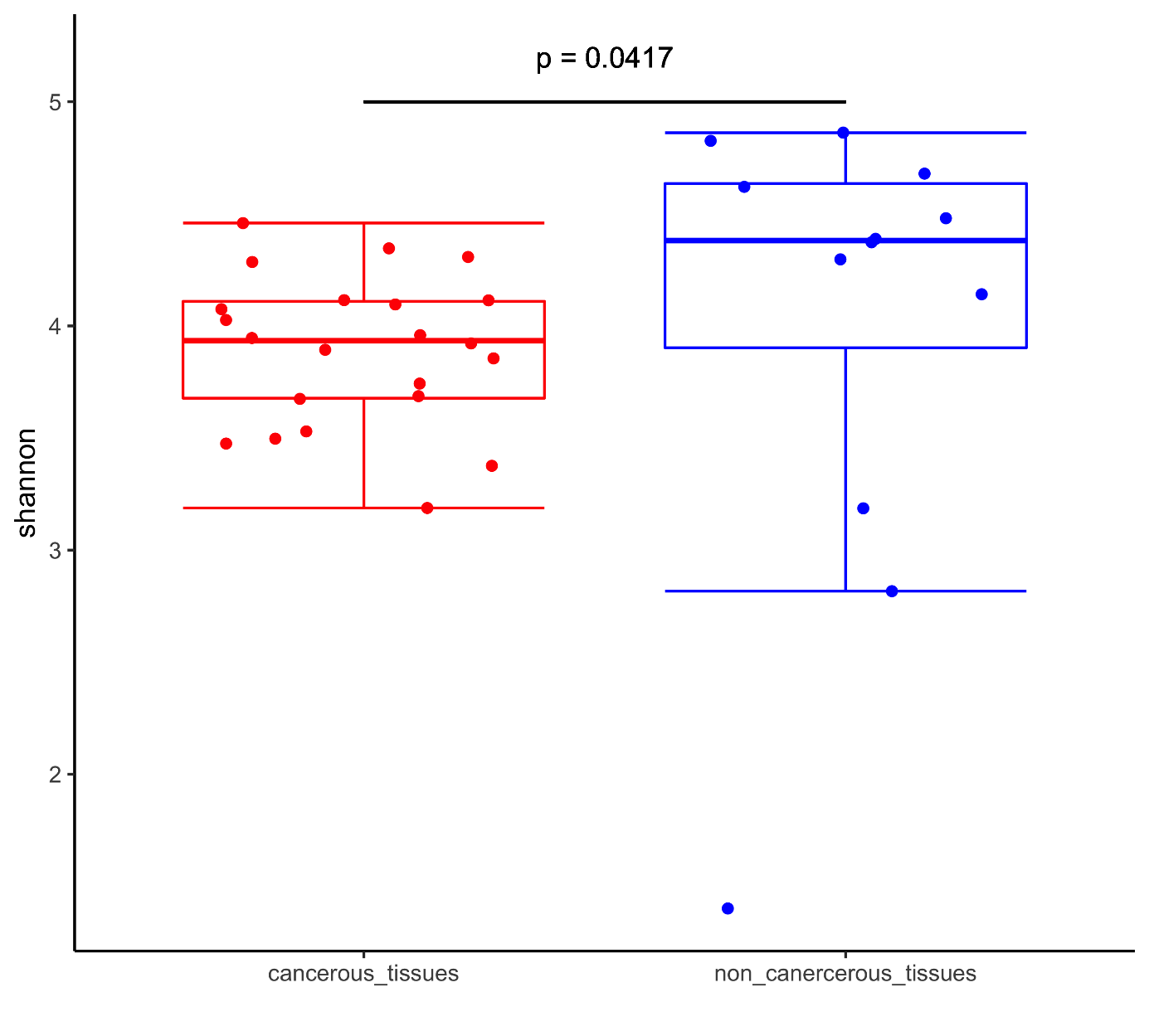


Figure.S1C


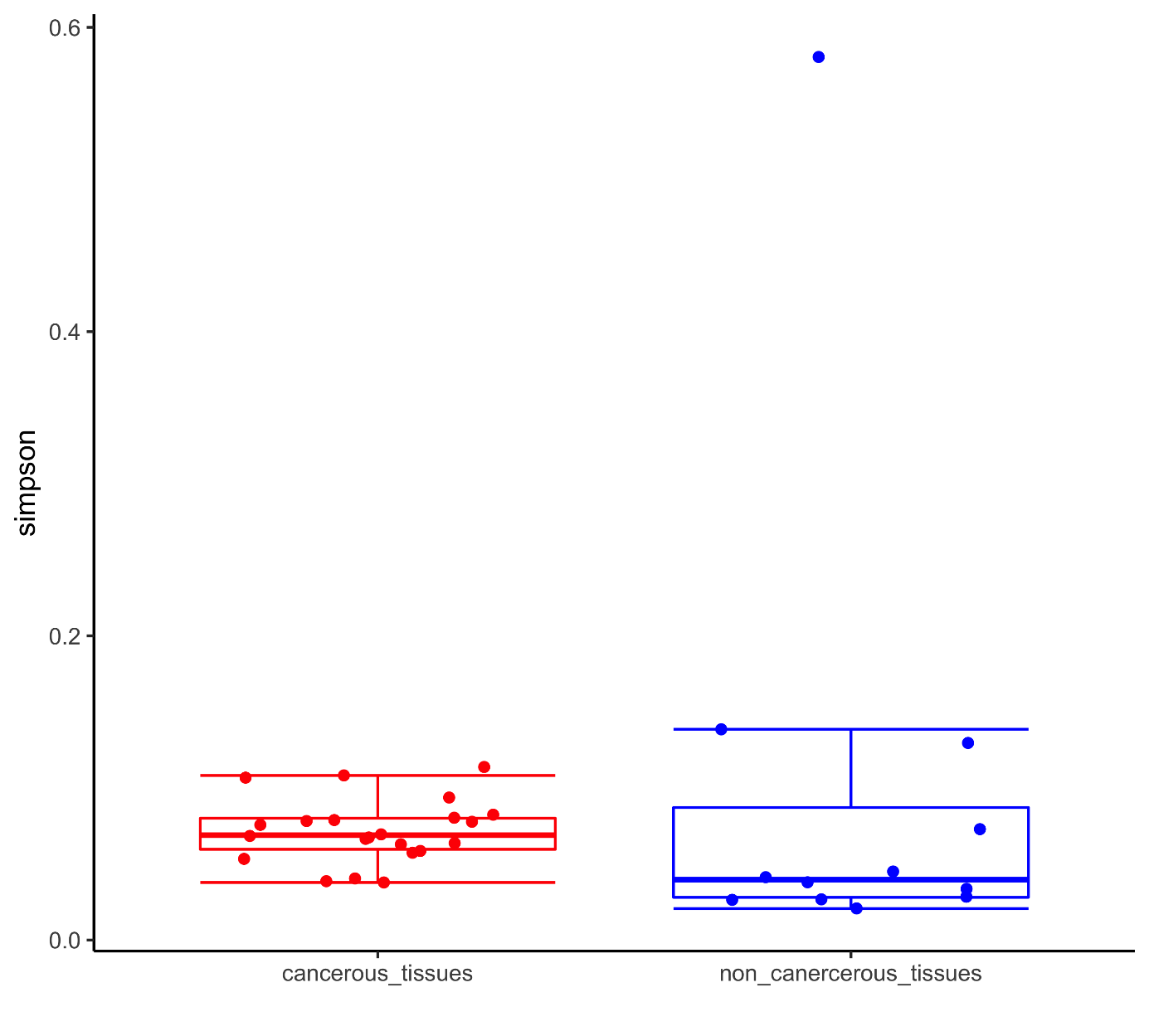


Figure.S1D


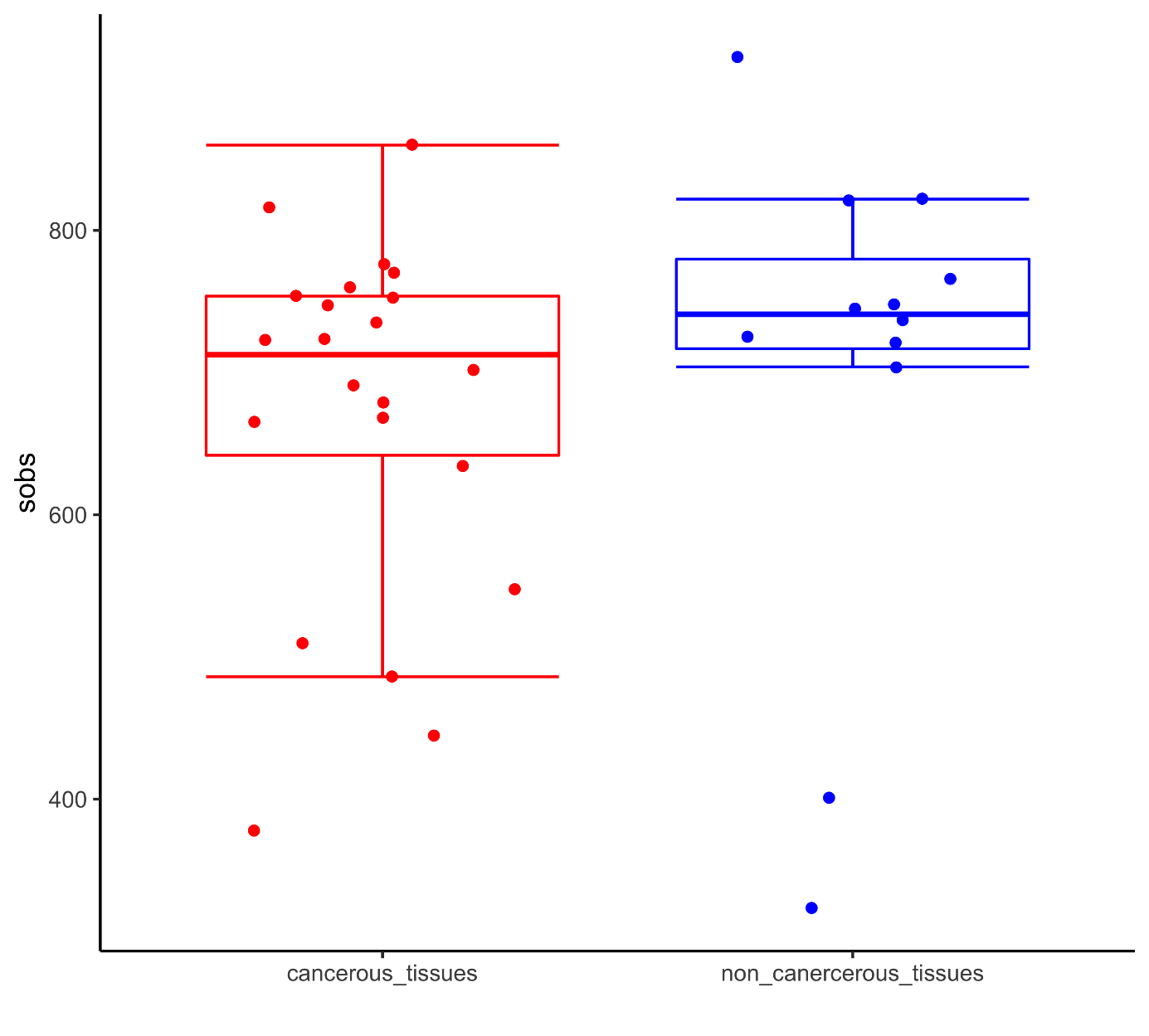


Figure.S1E

Supplement: Supplementary file 1 [file CAM4-8-6904-s001.docx]
